# In Situ Fluorescent Illumination of Microplastics in Water Utilizing a Combination of Dye/Surfactant and Quenching Techniques

**DOI:** 10.3390/polym14153084

**Published:** 2022-07-29

**Authors:** Doo Hong Park, Se Bin Oh, Sung Chul Hong

**Affiliations:** HMC, Department of Nanotechnology and Advanced Materials Engineering, Sejong University, Seoul 143-747, Korea; josephpark0916@gmail.com (D.H.P.); sevin6330@gmail.com (S.B.O.)

**Keywords:** microplastic, staining, fluorescent dye, surfactant, quenching

## Abstract

Although plastics have benefited our lives in terms of cost and convenience, the disposal of end-of-life plastics poses environmental problems, such as microplastics (MPs). Although the separation (e.g., filtration) and staining of MPs with fluorescent dye/solvent are generally accepted steps to observe MPs in an environmental matrix, in this study, an in situ selective fluorescent illumination of the MPs in water was attempted with the aid of surfactant. Nonpolar fluorescent dye in combination with surfactant affords nanometer-sized dye particles in water, which adsorb on MPs and penetrate the polymer matrix for effective staining and stable fluorescent behaviors. The effects of different staining parameters, including different dyes, surfactants, staining temperatures, staining times, dye/surfactant ratios, dye/MP ratios, and MP concentrations in aqueous solutions were investigated to better understand staining conditions. More interestingly, non-adsorbed free dye molecules in the staining solution were almost completely fluorescence-quenched by introducing the quenching agent, aniline, while the fluorescence intensity of the stained MP was maintained. By staining MPs with a dye/surfactant combination and subsequently quenching with aniline, in situ selective fluorescent illumination of the MPs in water was successfully achieved, which may eliminate the tedious separation/filtration procedure of MPs to accomplish the quick detection or monitoring of MPs.

## 1. Introduction

Plastic materials are extremely versatile due to their low density, low thermal/electrical conductivity, and resistance to corrosion, which allows them to be used in a wide range of applications, from food packaging to cutting edge devices [1,2]. The global production of plastics currently exceeds 320 million tons per year, over 40% of which is used as single-use packaging and results in waste [2,3]. Although plastics have benefited our lives in terms of cost and convenience, the disposal of end-of-life plastics poses environmental problems, since plastics progressively degrade and fragment into smaller pieces commonly known as microplastics (MPs) [2,3,4,5,6].

The Joint Group of Experts on the Scientific Aspects of Marine Environmental Protection defines MPs as plastic particles less than 5 mm in diameter [7]. Recently, Hartmann et al. proposed the size category of MPs of 1~1000 μm [8]. The characteristics of MPs enable them to persist in the environment, be transported over vast distances, bioaccumulate in the food web, and endanger the health of exposed organisms, including humans [3,9,10,11,12]. Over the last decade, MPs have emerged as novel pollutants that negatively affect terrestrial and especially aquatic environments [5,13,14,15].

Endeavors to observe/detect MPs in the environmental matrix are probably a first step to handle such a threat. The detection procedure of MP generally consists of separation (e.g., filtration), size categorization (e.g., sieving), purification (i.e., removal of organic residues), identification, and quantification [16,17,18,19,20,21]. For example, filtration is a common step to separate MPs from aqueous samples such as surface water, groundwater, marine water, and wastewater [17,18,22,23,24]. After the organic residues are removed, typically by oxidative methodology [18], MPs can be identified and quantified through spectroscopic observations, including optical observation, Fourier transform infrared spectroscopy, and Raman spectroscopy [4,25,26,27,28,29,30,31,32].

For these observations, staining MPs using fluorescent dyes is an essential step to visualize and identify MPs (Table S1, see Supplementary Materials) [33,34,35]. To facilitate incorporation into a nonpolar polymer matrix, lipophilic nonpolar dyes are generally employed. Nile red, which is an organic, hydrophobic, and photochemically stable heterocyclic compound, has been the most popular choice for this purpose (Table S1, see Supplementary Materials) [25,28,29,30,31,32]. Nile red is soluble in nonpolar media and exhibits good fluorescence intensity with high quantum yield.

Due to the low solubility of nonpolar dyes in water, researchers have generally used organic solvents to prepare staining solutions. Therefore, MPs must be separated from water and often dried before the staining procedures [31,32,36]. Acetone, chloroform, dimethyl sulfoxide, hexane, methanol, ethanol, and isopropyl alcohol are common solvents used to prepare the staining solutions (Table S1, see Supplementary Materials).

However, the use of organic solvents to stain MPs has distinct drawbacks, including the possibility of damaging MPs or filter materials, which may change the original characteristics of MPs. For example, because acetone is a solvent of polystyrene (PS), the size, shape, and perhaps number of recovered PS MPs may be changed by using acetone. In addition, the boiling temperature of the solvent is generally not so high (e.g., 56 °C for acetone), which may limit the upper staining temperature. More importantly, in recent years, there has been research interest regarding the real-time monitoring of MPs [37,38,39]. Dye staining procedures must also be adjusted to on-the-spot detection systems, where multi-step and time-consuming solvent-based staining methods are difficult to apply.

**Scheme 1:**
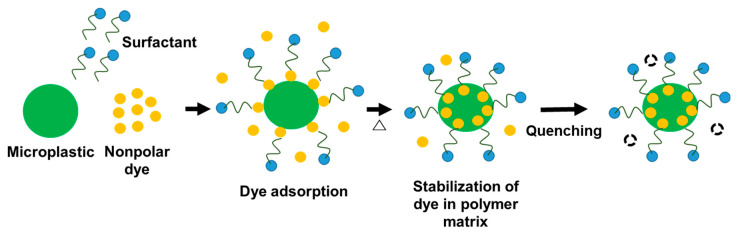
Schematic representation of the staining procedure of MPs in water with nonpolar dye and surfactant.

Herein, using surfactant, an in situ staining procedure of MPs in aqueous media is proposed. Surfactants are amphiphilic organic compounds composed of a hydrophobic tail and a hydrophilic head group [40]. With a certain amount of the selected surfactant, the nonpolar dye can be stabilized and solubilized by surfactant molecules in water [40,41,42]. The micelles from the surfactants are generally submicron in size, which may alleviate a concern of confusion with MPs (1~1000 μm, as defined by Hartmann et al.) [8,43]. The small size of the micelle also indicates its high surface area, which facilitates the adsorption of the dye in the micelle to the MP surface via the van der Waals interactions with additional dipole interactions to increase the staining efficiency (Scheme 1) [25,44]. At elevated temperatures, the polymer chain matrix becomes loosened, which enables the adsorbed dye to penetrate the polymer matrix [25,34]. The dye molecules become encapsulated in the polymer matrix by reducing the temperature to room temperature, which improves the fluorescent stability of the trapped dye in MPs even under subsequent harsh conditions, e.g., fluorescence quenching of free dye molecules (Scheme 1) [34]. To the best of these authors’ knowledge, there have been no examples of the direct staining of MPs in aqueous media using surfactants (Table S1, see Supplementary Materials).

## 2. Experimental

### 2.1. Materials

**Figure 1:**
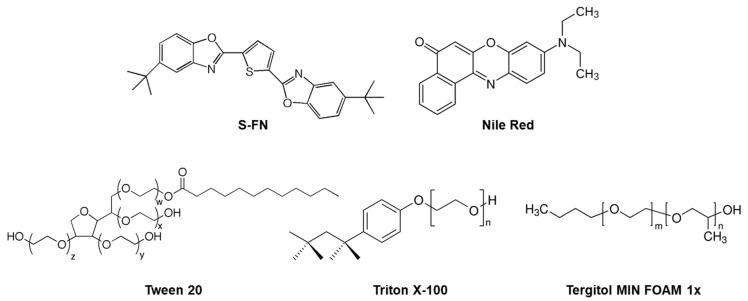
Chemical structures of dyes and surfactants in this study.

2,5-Bis(5-tert-butyl-2-benzoxazolyl)thiophene (S-FN, 99.0%, Yedam Chemical, Gimpo-si, South Korea) and Nile red (98.0%, Sigma–Aldrich, St. Louis, MO, USA) were employed as dyes and used without purification. Three representative commercially available nonionic surfactants, i.e., Tween 20 (Samchun, Seoul, South Korea), Triton X-100 (ForBioKorea, Seoul, South Korea), and Tergitol MIN FOAM 1x (Sigma–Aldrich, St. Louis, MO, USA), were purchased from the indicated suppliers. The chemical structures of the dyes and surfactants in this study are presented in Figure 1. Solvents including acetone (99.5%, Samchun, Seoul, South Korea), ethanol (94.5%, Samchun, Seoul, South Korea), and 1,4-dioxane (99.5%, Samchun, Seoul, South Korea) were used without purification. PS [weight average molecular weight (M_w_) = 35,000 g/mol], polypropylene (PP, M_w_ = 12,000 g/mol), and polyethylene (PE, M_w_ = 4000 g/mol) were purchased from Sigma–Aldrich (St. Louis, MO, USA). Polyethylene terephthalate (PET, granule size 3~5 mm) was purchased from Goodfellow (Coraopolis, PA, USA).

### 2.2. Preparation of Different MPs

A typical procedure to prepare MP samples is as follows: PS, PP, PE, and PET are crushed into microsized pieces using a blender (UNIX, UNB-A9100, Seoul, Korea). The crushed MPs are separated by size with testing sieves (Chunggye sieve; Gunpo-si, Korea). The sizes of the collected MPs are confirmed using an optical microscope (IF.2253-PLF, Euromex, The Netherlands), and the images are investigated with the ImageJ software program (National Institutes of Health, version 1.8.0_172). Unless noted, after sieving and size confirmation, MPs with sizes of 106~212 μm were used in subsequent experiments in this study.

### 2.3. MP Staining Procedures

A typical staining procedure of MPs is as follows: dye stock solution (typically, dye/surfactant = 1 g/L) is prepared by mixing 1.161 × 10^−5^ mole of dye (e.g., 5 × 10^−3^ g of S-FN) with 4.481 × 10^−3^ mole of surfactant (e.g., 5.5 g or 5 × 10^−3^ L of Tween 20) and stirred at 50 °C for 15 min. Separately, MP (~10 mg) is added to 10 mL of deionized water/ethanol mixed solution. Because the density values of the most common composition of MPs, i.e., PP, PE, and PS, are approximately 0.92, 0.93, and 1.09 g/cm^3^ (Table S2, see Supplementary Materials), respectively [2,36], the density of the aqueous staining solution is adjusted to approach the average density of the MP components (weighted by their approximated production proportion; ~35% for PP, ~56% for PE, and ~9% for PS) [5], ~0.95 g/cm^3^, by controlling the water/ethanol ratio (7.63/2.37 *v*/*v*) to better float MPs under occasional stirring. Then, a predetermined amount of dye/surfactant stock solution (e.g., 0.25 mL) is added to the MP solution for staining, followed by heating at 80 °C for 60 min by using an HS-R200 heating block (Humas Co., Ltd., Daejeon, South Korea).

### 2.4. Characterization

The size of the dye particles in water was observed by the dynamic light scattering method using a particle size analyzer (SZ-100, Horiba, Kyoto, Japan) at a scattering angle of 90° and a laser wavelength of 532 nm. For these experiments, the dye stock solution (dye/surfactant = 1 g/L) was diluted with 10 mL of water and stirred at 50 °C for 15 min to simulate the staining conditions. To take optical photographs of stained MPs, MPs were collected through filtration with F1002 grade Chmlab filter paper, repeatedly rinsed with deionized water, and illuminated with a UV Hand Lamp (VL-6LC, Vilber Lourmat, Collégien, France) at 254 nm for Nile red and 365 nm for S-FN. Fluorescence photographs of the stained MPs were collected by using a digital upright fluorescence microscope (Leica Microsystems, Wetzlar, Germany) at an excitation wavelength (325~375 nm)/emission wavelength (435~485 nm) for S-FN and an excitation wavelength (541~551 nm)/emission wavelength (565~605 nm) for Nile red. Fluorescence spectroscopy (FS-2, Scinco, Seoul, South Korea) was used to investigate the fluorescent emission spectra and relative fluorescence intensities of the stained MPs at their maximum absorption wavelengths (374 nm for S-FN and 520 nm for Nile red). For a rational comparison between the samples, a fixed quantity of stained MPs (150 mg) was employed for each fluorescence spectroscopy observation. At least three tests were repeated for each sample to determine the fluorescence intensity values with error bars.

## 3. Results and Discussion

**Figure 2:**
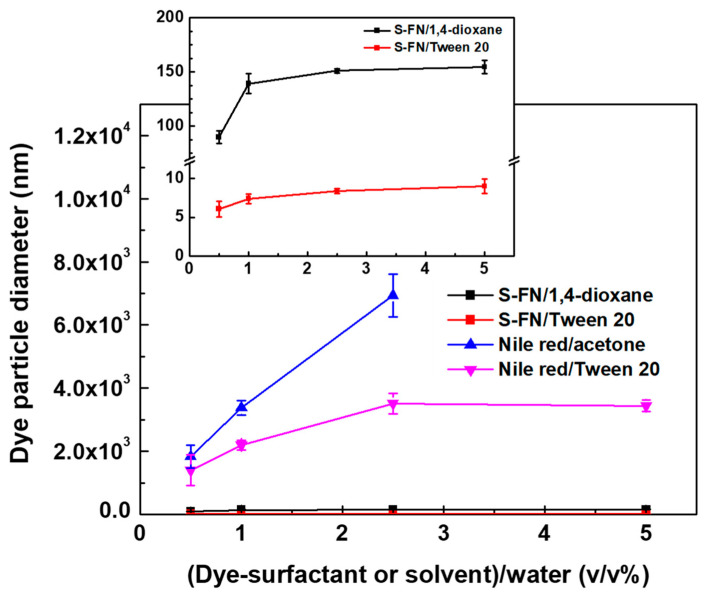
Size of dye particles with different dye and surfactant (or solvent) combinations at various concentrations in water (dye/surfactant or solvent = 1 g/L).

Most MPs are nonpolar materials with aliphatic chemical characteristics, which are generally difficult to stain in an aqueous solution due to their crystallinity, stereoregularity, and insolubility in water. Typical fluorescent dyes are also nonpolar unsaturated organic substances that absorb part of UV light and emit visible light to exhibit color. Although they have an affinity to MPs due to their hydrophobic nature, they are also insoluble in water, which limits the accessibility of the dye molecules to the polymer matrix in aqueous environments. For example, Nile red and S-FN are soluble in acetone and 1,4-dioxane, respectively, to form homogeneous solutions. Although acetone is miscible with water, Nile red/acetone added into water is no longer stable in a thermodynamic sense due to the nonpolar nature of Nile red and forms agglomerated/precipitated micrometer-sized dye particles (Nile red/acetone in Figure 2). At 5 vol% Nile red/acetone in water, the dye particles are too large to be detected by the particle size analyzer (out of the detection limit of the analyzer). This large dye particle size is obviously not acceptable for the staining procedure of MPs, since the agglomerated dye particles can be confused with MPs and result in the overcounting of MPs. The staining of MPs can also be ineffective due to poor adsorption of the large dye particles on MPs, which limits the access of Nile red molecules to MPs for staining. Similarly, although 1,4-dioxane is miscible with water, S-FN/1,4-dioxane provided dye particles with sizes of 100~150 nm (inset of Figure 2).

In contrast, the introduction of a surfactant, i.e., Tween 20, dramatically reduced the dye particle sizes in water. For Nile red, the size of the dye particles was reduced to ~3 μm at 2.5 vol% Nile red/Tween 20 in water (Figure 2). For S-FN, the size of the dye particle was smaller than 10 nm in the presence of Tween 20, which likely suggests the formation of micelles. Because the critical micelle concentration (CMC) of Tween 20 in water is 5.5 × 10^−3^ vol% [45], all dye solution concentrations of the samples in Figure 2 were well above the CMC, which suggests the solubilization of S-FN through the formation of dye solubilized micelles of Tween 20 [40]. Since sizes > 1 μm are generally targeted during the MP detection, the results well support the advantages of using surfactants (i.e., Tween 20 in this case). In addition, the results suggest that S-FN is preferred over Nile red in terms of the solubilization of the dyes in water.

Compared with Nile red, the improved solubilization of S-FN in the presence of Tween 20 also resulted in better fluorescent behavior of the resulting stained MPs. As shown in Figure S1 (see Supplementary Materials), MPs stained with S-FN/Tween 20 (set this fluorescent intensity of MP as a reference value, 1) had the highest relative fluorescence intensity among the fluorescence intensity values of MPs from other dye/solvent or surfactant combinations. The MPs stained with Nile red/acetone had the lowest relative fluorescent intensity among the MPs, likely due to the poor solubilization of Nile red to form dye agglomerates (Figure 2). In any case, compared with the usage of solvents (e.g., 1,4-dioxane for S-FN and acetone for Nile red), the incorporation of Tween 20 improved the staining efficiency, which suggests the advantages of surfactant to solubilize dyes and effectively stain MPs.

**Figure 3:**
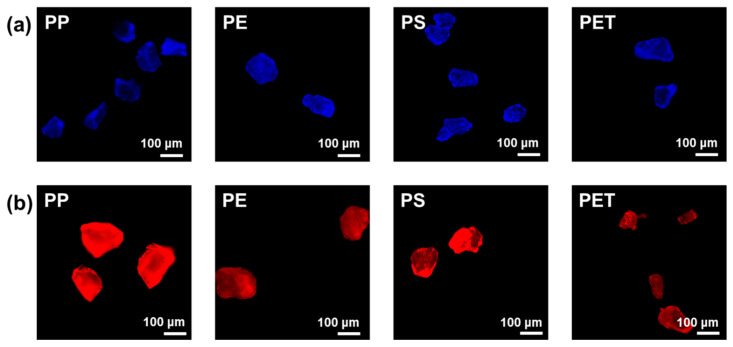
Fluorescence photographs of MPs with different compositions stained with S-FN (**a**) and Nile red (**b**). Staining conditions: surfactant = Tween 20, dye/surfactant = 1 g/L, dye/MP = 2.5 wt%, MP/aqueous solution = 1 g/L, staining time = 60 min, staining temperature = 80 °C.

The effect of different amounts of S-FN on the size of the dye particle is shown in Figure S2 (see Supplementary Materials). Increasing the amount of S-FN to 2 g/L of S-FN/Tween 20 resulted in slightly larger particles than that with 1 g/L of S-FN/Tween 20. For the 4 g/L S-FN/Tween 20 solution, large dye agglomerates with sizes of 2~6 μm were observed, naturally due to the excessive amount of S-FN. Based on these observations, 1 g/L of dye/surfactant at 2.5 vol% in water was adopted as a standard composition of dye solution for subsequent staining experiments. No specific differences in the particle sizes of S-FN with different surfactants were observed (Figure S3, see Supplementary Materials). Figure 3 demonstrates successful staining of various MPs by using S-FN/Tween 20 (Figure 3a) or Nile red/Tween 20 (Figure 3b) under the indicated staining conditions, which exhibits clear fluorescence emission of the stained MPs.

**Figure 4:**
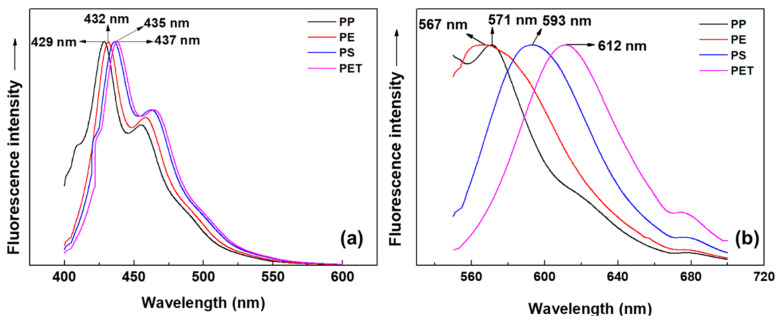
Normalized fluorescence spectra of S-FN (**a**) and Nile red (**b**) in stained MPs. Staining conditions: surfactant = Tween 20; dye/surfactant = 1 g/L; dye/MP = 2.5 wt%; MP/aqueous solution = 1 g/L; staining time = 60 min; staining temperature = 80 °C.

Figure 4 shows the emission fluorescence spectra of MPs stained with S-FN and Nile red in the presence of Tween 20. MPs stained with S-FN exhibited fluorescence spectra with maximum emission wavelengths of 429~437 nm, depending on the MP compositions. The emission wavelength values of S-FN were quite consistent and similar, which suggests the orthochromatic characteristics of S-FN. Regardless of the MP compositions and staining temperatures, the constant fluorescent emission behaviors of S-FN are also observed as blue MPs in optical photographs in Figure S4 (under 365 nm illumination with UV Hand Lamp, see Supplementary Materials).

In contrast, the maximum emission wavelengths of MPs stained with Nile red were quite diversified according to the compositions of MPs (567~612 nm, Figure 4b). For example, the maximum emission wavelength of Nile red in stained PE was 567 nm, while those of Nile red in PP, PS, and PET were 571 nm, 593 nm, and 612 nm, respectively (Figure 4b). The fluorescence emission behaviors also resulted in yellow, orange, and red colors of PP, PE, and PS MPs, respectively, in optical observations (Nile red in Figure S4, under 254 nm illumination with UV Hand Lamp, see Supplementary Materials). The results suggest the solvatochromic characteristics of Nile red in different types of MPs, as also reported in other literature [25,46,47,48,49]. The solvatochromic properties of Nile red indicate different staining colors with different microenvironmental polarities. With other surfactants, e.g., Tergitol MIN FOAM 1x, similar behaviors were observed (Figure S5, see Supplementary Materials), which suggests no significant effect of different surfactants on the emission characteristics of the dyes. For the simple and efficient detection of MPs at a fixed wavelength, in this study, the orthochromatic characteristics of S-FN were preferred. In addition, as shown in Table S3 (see Supplementary Materials), S-FN was considerably cheaper than Nile red and other renowned dyes due to its wide application as a fluorescent brightening agent in industry, which enabled us to adopt S-FN as a main dye to stain MPs in subsequent studies.

To confirm the fluorescence stability of the stained MPs, the staining solution was removed by filtration after the staining of MPs, and the resulting stained MPs were redispersed in water/ethanol aqueous solution (7.63/2.37 *v*/*v*, density ~ 0.95 g/cm^3^). As shown in Figure S6 (see Supplementary Materials), the stained MPs with S-FN exhibited clear blue fluorescence color in aqueous solution under illumination with UV Hand Lamp at 365 nm, which supports the successful incorporation of S-FN into the polymer matrix of MPs for their staining. Even after 10 days, a clear blue fluorescence color was still observed (Figure S6, see Supplementary Materials), which supports the successful trapping and stabilization of S-FN in the polymer matrix of MPs.

**Figure 5:**
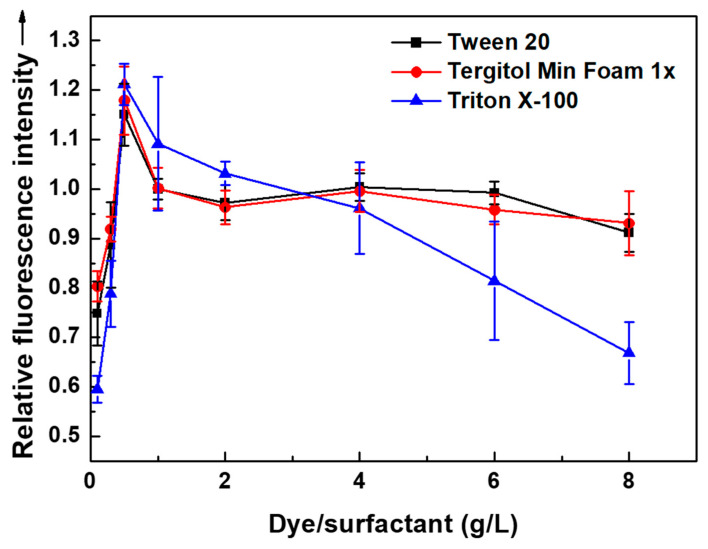
Relative fluorescence intensity of PP MPs stained with different surfactants at different dye/surfactant ratios. Staining conditions: dye = S-FN; dye/MP = 2.5 wt%; MP/aqueous solution = 1 g/L; staining time = 60 min; staining temperature = 80 °C.

The effects of different surfactants and dye/surfactant ratios were investigated by observing the relative fluorescence intensity of PP MPs stained with S-FN (Figure 5). In the dye/surfactant range of 0~1 g/L, relatively higher fluorescent intensities were observed with a higher dye/surfactant ratio, which was attributed to the increased amount of free dye available to access the MPs [50]. Conversely, further increased dye/surfactant ratios above 1 g/L resulted in slightly lower fluorescent intensities of MPs, likely due to an inefficient solubilization of dye with a reduced amount of surfactant (Figure 5) [50]. Unlike Triton X-100, the fluorescent intensities of S-FN-stained MPs with Tween 20 and Tergitol MIN FOAM 1x were relatively consistent regardless of the amount of surfactant, which suggests the stable and superior solubilization capability of Tween 20 and Tergitol MIN FOAM 1x for S-FN. Considering the results from Figure 5 and Figure S3, Tween 20 was selected as a preferred surfactant for S-FN for reliable experiments.

**Figure 6:**
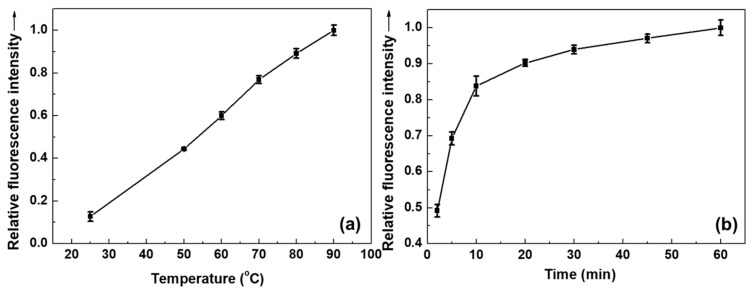
Relative fluorescence intensity of PP MPs stained with S-FN/Tween 20 at different staining temperatures (**a**) and times (**b**). Staining conditions: dye/surfactant = 1 g/L; dye/MP = 2.5 wt%; MP/aqueous solution = 1 g/L. The staining time is 60 min for (**a**), and the staining temperature is 80 °C for (**b**).

The effects of different parameters, such as the temperature, time, dye/MP ratios, and MP/aqueous solution ratios on the staining characteristics of MPs with S-FN/Tween 20 were investigated. Heating the aqueous MP staining mixture can increase the dye’s affinity for MPs [34]. As shown in Figure 6a, higher relative fluorescence intensities of MPs were observed with higher staining temperatures. At higher temperatures, the polymer chains of MPs likely became loosened, which enabled S-FN to penetrate the polymer matrix more easily. As a result, more S-FN was encapsulated in MPs, so they exhibited higher fluorescent intensities [25]. However, as shown in Figure S4 and Table S2 (see Supplementary Materials), MPs may agglomerate with each other due to the fusion above individual glass transition temperatures or melting temperatures, which should be avoided to rationally detect MPs. For example, above the generally accepted melting temperature of PE (120 °C, Table S2, see Supplementary Materials), agglomerations of PE MPs were observed (Figure S4, see Supplementary Materials). The average glass transition temperature of polystyrene is ~100 °C (Table S2, see Supplementary Materials), so PS MPs agglomerated above the staining temperature of 100 °C. Therefore, in this study, the upper limit of the staining temperature was set to 90 °C to avoid the MP fusion.

Due to similar reasons, i.e., penetration of more S-FN into the polymer matrix, a longer staining time resulted in higher fluorescent intensities of MPs at a fixed staining temperature (80 °C, Figure 6b). However, the relative fluorescent intensities of MPs levelled off after 10 min of staining. The results indicate that a short staining time (~10 min) was sufficient to ensure a high fluorescent intensity of the stained MPs. The results likely stemmed from the limited penetration of S-FN mainly into the surface layer of PP MP. Because the representative melting temperature of PP is 156 °C (Table S2, see Supplementary Materials), the staining temperature (80 °C) was far below the melting temperature, and the diffusion of S-FN was limited to the surface layer of PP MPs.

**Figure 7:**
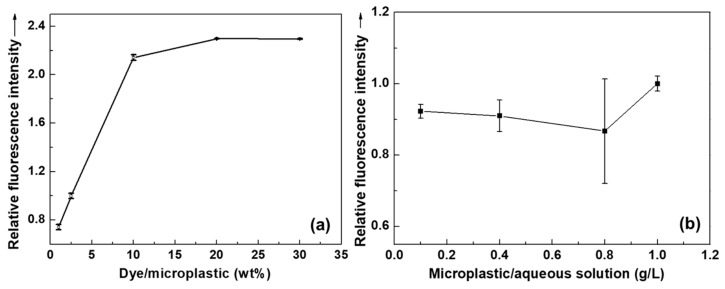
Relative fluorescence intensity of PP MPs stained with different dye/MP (**a**) and MP/aqueous solution (**b**) ratios. Staining conditions: dye = S-FN; surfactant = Tween 20; dye/surfactant = 1 g/L; staining time = 60 min; staining temperature = 80 °C. MP/aqueous solution is 1 g/L for (**a**), and dye/MP is 2.5 wt% for (**b**).

At a fixed dye/surfactant ratio of 1 g/L, the addition of more dye to MP naturally increased the relative fluorescent intensities of stained MPs due to higher staining probabilities in the presence of more dye (Figure 7a). However, above 10 wt% dye/MP, the relative fluorescent intensities of the stained MPs did not further increase, likely due to the limited solubility of the dye (at a fixed dye/surfactant ratio) or the self-quenching phenomena of the dye [51]. In the presence of excess dye, the emitted radiation may be reabsorbed by the adjacent dye or transmitted to the surrounding polymer matrix as thermal vibration energy, which may limit the overall fluorescent intensities [52].

More than 18,000 particles of MPs are reportedly often found in 1 L of wastewater [53]. Assuming that the shape and diameter of the MPs are spherical and 159 μm (median value of 106~212 μm of this study), respectively, this concentration of PP MPs (i.e., 18,000 MP particles/L) corresponds to 3.5 × 10^−2^ g/L of MP [density of PP = 0.92 g/cm^3^, volume of MP = (1/6) × π × d^3^] [54]. Figure S7 shows the fluorescent behavior of PP MPs stained with S-FN/Tween 20 at this concentration. The PP MPs were collected through filtration (Figure S7a) or redispersed in aqueous solution (Figure S7b) for observations (illuminated by UV Hand Lamp at 365 nm), which clearly demonstrates the successful staining of PP MPs with the dye/surfactant combination even at this low MP concentration.

To semi-quantitatively characterize stained MPs by using FS-2 fluorescence spectroscopy, 150 mg of MP sample is required. Practically, an MP/aqueous solution concentration of 3.5 × 10^−2^ g/L is too low to collect such a quantity of MPs. In addition, to stain MPs viably and effectively, the MP-containing aqueous sample can be concentrated to some degree prior to staining by partial removing of water. In this context, the staining behavior of the MP aqueous solution in the MP/aqueous solution range of 0.1~1.0 g/L was checked (~10 times concentrated than 3.5 × 10^−2^ g/L). As shown in Figure 7b, the relative fluorescent intensities of stained PP MPs determined by FS-2 were almost constant regardless of the tested MP concentration, which again supports the good staining capability of the S-FN/Tween 20 combination in this study.

Figure S8 (see Supplementary Materials) shows the effect of different sizes of MPs stained with S-FN/Tween 20 on their fluorescence behaviors. All MPs exhibited strong fluorescence intensities. Interestingly, the larger MPs exhibited slightly higher fluorescent intensities, although the reason remains unclear.

**Figure 8:**
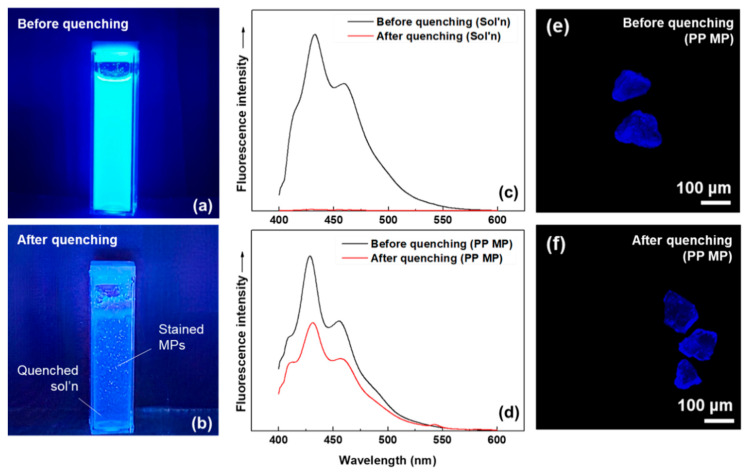
Optical photographs of the aqueous staining solution with PP MPs before (**a**) and after (**b**) quenching with aniline illuminated by UV Hand Lamp at 365 nm. Fluorescence spectra of the aqueous staining solution (without MPs, (**c**)) and stained PP MPs (**d**) before and after quenching with aniline. Fluorescence photographs of stained PP MPs before (**e**) and after (**f**) quenching with aniline. Staining conditions: dye = S-FN; surfactant = Tween 20; dye/surfactant = 1 g/L; dye/MP = 2.5 wt%; MP/aqueous solution = 1 g/L; staining time = 60 min; staining temperature = 80 °C. Fluorescence quenching condition: 0.25 M aniline, room temperature.

As mentioned in the introduction, research interest regarding the real-time and on-the-spot detection of MPs has increased. To realize this detection, in situ fluorescence quenching of non-adsorbed free dye molecules in staining solution is proposed in this study to selectively illuminate stained MPs without filtration or drying (final step of Scheme 1). To check the feasibility, 0.25 M aniline was introduced into the aqueous staining solution in the presence of MPs, and their fluorescence behavior was observed. Aniline was specifically adopted as a quenching agent based on the literature studies [55,56]. As shown in Figure 8, immediately after the introduction of aniline, the fluorescence intensity of the free dye in the staining solution almost completely disappeared (Figure 8a versus Figure 8b, and Figure 8c). However, the fluorescence intensity of the stained MP was well maintained regardless of the addition of aniline, likely due to the encapsulation and trapping effect of the dye in the polymer matrix to protect the dye from quenching (Figure 8b,d). The results were also well evidenced by the clear fluorescent emission of collected PP MPs before and after quenching (Figure 8e,f). In other words, in the presence of MPs, the fluorescent activity of the free non-adsorbed dye in solution was selectively eliminated by introducing the quenching agent aniline and leaving the selective fluorescent illumination of the stained MPs (Figure 8a versus Figure 8b). The results suggest that the staining solution may not have to be removed after the staining procedures to observe MPs, which may dramatically simplify the detection system of MPs by eliminating tedious separation/filtration/drying procedures to collect MPs. Application of the developed methodology to on-site monitoring of MPs will be the content of our next research.

## 4. Conclusions

In this study, with the aid of a surfactant, in situ staining of MPs in aqueous media is proposed. Despite the nonpolar nature of fluorescent dyes, the introduction of the surfactant dramatically improved the solubility of the dyes in water, as evidenced by the reduced dye particle sizes (~10 nm for S-FN/Tween 20) and afforded the efficient and successful fluorescent staining of MPs. The S-FN-stained MPs exhibited a clear blue fluorescence color with long-term stability, supporting successful incorporation of S-FN into the polymer matrix of MPs. MPs stained with S-FN exhibited orthochromatic characteristics and relatively consistent fluorescent emission spectra with maximum emission wavelengths of 429~437 nm regardless of the MP and surfactant compositions. With S-FN/Tween 20, the study on different staining parameters, such as the temperature, time, and dye/MP ratios on the staining characteristics of MPs indicated that 90 °C, >10 min, and ~10 wt% dye/MP, were the optimal staining conditions. Staining of PP MP with S-FN/Tween 20 was possible for various MP/aqueous solution concentrations of 3.5 × 10^−2^~1.0 g/L, which supports the good staining capability. Fluorescence quenching of non-adsorbed free dye molecules in staining solution was successfully accomplished by introducing aniline, while the fluorescent intensity of stained MP was well maintained due to the encapsulation and protecting effect of the dye by the polymer matrix. Through the staining and quenching procedure, in situ selective fluorescent illumination of the stained MPs in the staining solution was successfully achieved, which may eliminate the tedious separation/filtration procedure to detect/monitor MPs.

## Data Availability

The data presented in this study are available in within this article.

## Supplementary Materials

The following supporting information can be downloaded at: https://www.mdpi.com/article/10.3390/polym14153084/s1, Supplementary Materials associated with this article can be found as a separate file: 3 Tables and 8 Figures. Table S1, Examples of MP staining methods; Table S2, Representative density and fusion temperature values of common MPs; Table S3, Comparison between recent prices of dye candidates to stain MPs; Figure S1, Relative fluorescence intensity of PP-MPs stained with S-FN and Nile red with Tween 20 and different solvents. Staining conditions: dye/surfactant or solvent = 1 g/L; dye/MP = 2.5 wt%; MP/water solution = 1 g/L; staining time = 60 min; staining temperature = 80 °C. Figure S2, Size of the dye particles with different dye/surfactant ratios at various S-FN/Tween 20 concentrations in water; Figure S3, Size of the dye particles with different surfactants at various S-FN concentrations in water (dye/surfactant = 1 g/L); Figure S4, Optical photographs of different stained MPs at different staining temperatures (illumination by UV Hand Lamp at 254 nm for MPs stained with Nile red and 365 nm for MPs stained with S-FN). Staining conditions: surfactant = Tween 20; dye/surfactant = 1 g/L; dye/MP = 2.5 wt%; MP/aqueous solution = 1 g/L; staining time = 60 min. Figure S5, Normalized fluorescence spectra of S-FN (a) and Nile red (b) in stained MPs. Staining conditions: surfactant = Tergitol MIN FOAM 1x; dye/surfactant = 1 g/L; dye/MP = 2.5 wt%; MP/aqueous solution = 1 g/L; staining time = 60 min; staining temperature = 80 °C. Figure S6, Optical photographs of stained MPs after removing the staining solution and redispersing the MPs in a water/ethanol solution (illuminated by UV Hand Lamp at 365 nm). Staining conditions: dye = S-FN; surfactant = Tween 20; dye/surfactant = 1 g/L; dye/MP = 2.5 wt%; MP/aqueous solution = 1 g/L; staining time = 60 min; staining temperature = 80 °C. Figure S7, Optical photographs of PP MPs stained at an MP/aqueous solution concentration of 3.5 × 10^−2^ g/L: Filtered PP MPs (a) and redispersed PP MPs in aqueous solution (b) after staining (illuminated by UV Hand Lamp at 365 nm). Staining conditions: dye = S-FN; surfactant = Tween 20; dye/surfactant = 1 g/L; dye/MP = 2.5 wt%; MP/aqueous solution = 3.5 × 10^−2^ g/L; staining time = 60 min; staining temperature = 80 °C. Figure S8, Fluorescence spectra of PP MPs with different sizes stained with S-FN/Tween 20. Staining conditions: dye/surfactant = 1 g/L; dye/MP = 2.5 wt%; MP/aqueous solution = 1 g/L; staining time = 60 min; staining temperature = 80 °C. References [2,5,18,28,29,30,31,32,33,34,36,48,57,58,59,60,61,62,63,64,65,66] are cited in the Supplementary Materials.
