# Supplementary material for: In Situ Fluorescent Illumination of Microplastics in Water Utilizing a Combination of Dye/Surfactant and Quenching Techniques
Source: Polymers (Basel). 2022 Jul 29;14(15):3084. doi: 10.3390/polym14153084 (PMC9370827; doi:10.3390/polym14153084)
Supplement: Supplementary file 1 [file polymers-14-03084-s001.zip › polymers-1816285-supplementary.pdf]

Supplementary Material: In Situ Fluorescent Illumination of Microplastics in Water Utilizing a Combination of Dye/Surfactant and Quenching Techniques

Doo Hong Park, Se Bin Oh and Sung Chul Hong *

Table S1. Examples of MP staining methods.

Polymer type [a]	shape	Size range (μm)	Dye	Solvent	Excitation wavelength (nm)	Emission wavelength (nm)	Reference
PS	Fragment, sphere	13.5 ~ 300	iDye Poly(Jacquard)	Deionized water	531	593	[1]
	Fragment	-	Nile red	Acetone	450~510	529	[2]
	Fragment Granule	300 ~ 5000	Nile red	Chloroform Acetone n-Hexane	365	445	[3]
	Fragment	63 ~ 630	Nile red	Chloroform	365	445	[4]
	Fragment	24 ~ 196	Nile red	Methanol	460	525	[5]
	Bead	0.05 ~ 0.1	Nile red	Methanol	623	660	[6]
	Fragment	~ 100	Nile red	Methanol	460	543	[7]
	Fragment	~ 100	FITC	Methanol	488	-	[7]
	Fragment	~ 100	Safranine T	Methanol	520	-	[7]
	Fragment	-	DAPI	Water	355 ~ 405	420 ~ 480	[8]
	Fragment	100 ~ 500	Rhodamine B	Ethanol	450 ~ 490	515 ~ 565	[9]
LDPE	Fragment	100 ~ 500	iDye Poly(Jacquard)	Deionized water	531	593	[1]
	Fragment Granule	300 ~ 5000	Nile red	Chloroform Acetone n-Hexane	365	445	[3]
	Fragment		Nile red	Acetone	440~520	535	[10]
	Fragment	~ 300	Nile red	n-Hexane	534 ~ 558	515 ~ 565, 590 ~	[11]
HDPE	Sphere	10 ~ 90	iDye Poly(Jacquard)	Deionized water	531	593	[1]
	Fragment Granule	300 ~ 5000	Nile red	Chloroform Acetone n-Hexane	365	445	[3]
	Fragment		Nile red	Acetone	440~520	535	[10]
	Fragment	300 ~ 1000	Nile red	Methanol	460	525	[12]
	Fragment	80 ~ 200	Nile red	n-Hexane	534 ~ 558	515 ~ 565, 590 ~	[11]
PP	Fragment	500 ~ 4000	iDye Poly(Jacquard)	Deionized water	531	593	[1]
	Fragment	-	Nile red	Acetone	450~510	529	[2]
	Fragment Granule	300 ~ 5000	Nile red	Chloroform Acetone n-Hexane	365	445	[3]
	Fragment		Nile red	Acetone	440~520	535	[10]
	Fragment	63 ~ 630	Nile red	Chloroform	365	445	[4]
	Fragment	300 ~ 1000	Nile red	Methanol	460	525	[12]
	Fragment	20 ~ 130	Nile red	Methanol	460	525	[5]
	Fragment	~ 300	Nile red	n-Hexane	534 ~ 558	515 ~ 565, 590 ~	[11]
	Fragment	-	DAPI	Water	355 ~ 405	420 ~ 480	[8]
	Fragment	400 ~ 1000	Rhodamine B	Ethanol	450 ~ 490	515 ~ 565	[9]
PET	Fragment,	30 ~ 5000	iDye Poly(Jacquard)	Deionized	531	593	[1]

	fiber			water		
	Fiber	300 ~ 5000	Nile red	Chloroform<br>Acetone<br>n-Hexane	365	445	[3]
	Fragment	-	Nile red	Acetone	450~510	529	[2]
	Fiber	63 ~ 630	Nile red	Chloroform	365	445	[4]
	Fragment	~ 300	Nile red	Methanol	460	525	[5]
	Fragment	-	Nile red	n-Hexane	365	445	[11]
	Fragment	~ 200	Nile red	Methanol	460	543	[7]
	Fragment	~ 200	FITC	Methanol	488	-	[7]
	Fragment	~ 200	Safranine T	Methanol	520	-	[7]
PVC	Fragment	50 ~ 300	iDye Poly(Jacquard)	Deionized water	531	593	[1]
	Fragment	80 ~ 148	Nile red	Methanol	460	525	[5]
	Fragment		Nile red	n-Hexane	534 ~ 558	515 ~ 565, 590 ~	[11]
	Fragment	~ 100	Nile red	Methanol	460	543	[7]
	Fragment	~ 100	FITC	Methanol	488	-	[7]
	Fragment	~ 100	Safranine T	Methanol	520	-	[7]
	Fragment	-	DAPI	Water	355 ~ 405	420 ~ 480	[8]
	Fragment	50 ~ 200	Rhodamine B	Ethanol	450 ~ 490	515 ~ 565	[9]
PAN	Fiber	20 ~ 3000	iDye Poly(Jacquard)	Deionized water	531	593	[1]
	Fragment	63 ~ 630	Nile red	Chloroform	365	445	[4]
PE	Fragment	-	Nile red	Acetone	450~510	529	[2]
	Fragment	63 ~ 630	Nile red	Chloroform	365	445	[4]
	Fragment	40 ~ 48	Nile red	Methanol	460	525	[5]
	Fragment	40 ~ 46	Nile red	Methanol	460	525	[13]
	Fragment	100 ~ 300	Rhodamine B	Ethanol	450 ~ 490	515 ~ 565	[9]
Nylon 6	Fragment	-	Nile red	Acetone	450~510	529	[2]
	Fragment	63 ~ 91	Nile red	Methanol	460	525	[5]
PA	Fragment	63 ~ 630	Nile red	Chloroform	365	445	[4]
	Fiber	-	Nile red	n-Hexane	365	445	[11]
	Fiber	-	DAPI	Water	355 ~ 405	420 ~ 480	[8]
PC	Fragment	94 ~ 169	Nile red	Methanol	460	525	[5]
PU	Fragment	71 ~ 154	Nile red	Methanol	460	525	[5]
	Fragment	~ 100	Nile red	n-Hexane	534 ~ 558	515 ~ 565, 590 ~	[11]
	Fragment	50 ~ 300	Rhodamine B	Ethanol	450 ~ 490	515 ~ 565	[9]
EPS	Fragment	~ 300	Nile red	n-Hexane	534 ~ 558	515 ~ 565, 590 ~	[11]

[a] Abbreviations of polymers: Polystyrene, PS; Polyethylene, PE; high-density polyethylene, HDPE; low-density polyethylene, LDPE; polypropylene, PP; poly(ethylene terephthalate), PET; poly(vinyl chloride), PVC; polyacrylonitrile, PAN; polyamide, PA; polycarbonate, PC; polyurethane, PU; expanded polystyrene, EPS.

Table S2. Representative density and fusion temperature values of common MPs [1,14-22].

Type	Composition	Density range (g/cm^3)	Representative density (average, g/cm^3)	Thermal event for fusion [a]	Fusion temperature (°C)	Representative fusion temperature (average, °C)
PP	isotactic PP	0.90 ~ 0.91	0.92	T_m	~ 186	156
	syndiotactic PP	0.90 ~ 0.95		T_m	125 ~ 130	
PE	High density PE (HDPE)	0.94 ~ 0.96	0.93	T_m	125	120
	Low density PE (LDPE)	0.91 ~ 0.93		T_m	130	
	Linear low density PE (LLDPE)	0.91 ~ 0.93		T_m	105 ~ 125	
	Very low density PE (VLDPE)	0.90		T_m	110	
PS	General purpose PS	1.03 ~ 1.05	1.09	T_g	90 ~ 100	100
	Styrene-acrylonitrile copolymer (SAN)	1.07 ~ 1.09		T_g	103 ~ 109	
	Acrylonitrile-butadiene-styrene copolymer (ABS)	1.01 ~ 1.8		T_g	104	

[a] T_m = melting temperature, T_g = glass transition temperature.

Table S3. Comparison between recent prices of dye candidates to stain MPs [a].

Dye	Source	Cost (KRW/g)	Cost (USD/g)
Nile red	Sigma Aldrich	981,976	792.28
S-FN	Yedam chemical	149	0.12
Fluorescein isothiocyanate (FITC)	Sigma Aldrich	369,214	297.89
4,5,6,7-Tetrachloro-2′,4′,5′,7′-tetraiodofluorescein disodium salt (Rose Bengal)	Sigma Aldrich	67,512	54.47

[a] 1 USD = 1,239 KRW on April 22th, 2022.

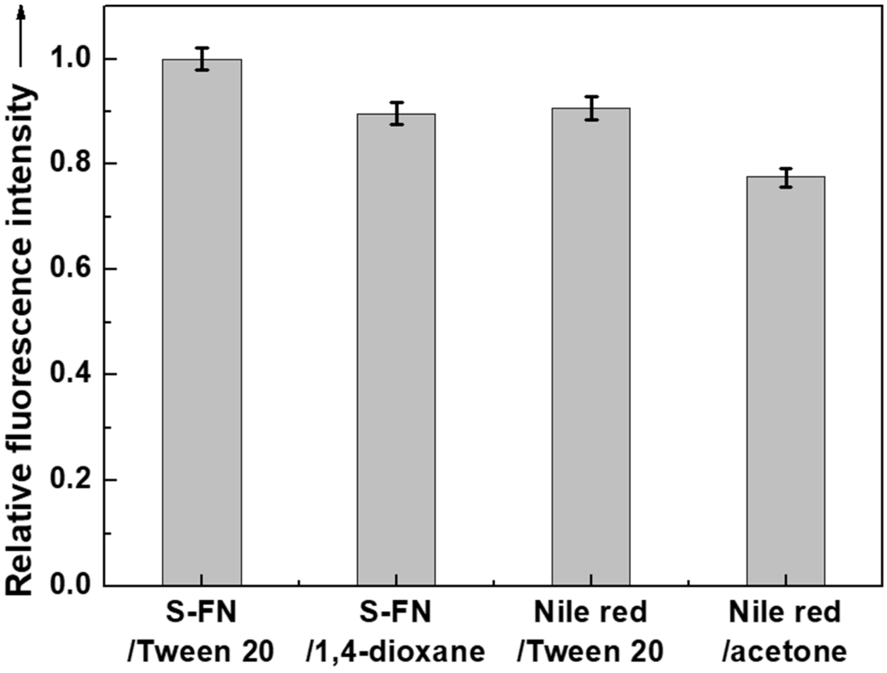

Figure S1. Relative fluorescence intensity of PP-MPs stained with S-FN and Nile red with Tween 20 and different solvents. Staining conditions: dye/surfactant or solvent = 1 g/L, dye/MP = 2.5 wt%, MP/water solution = 1 g/L, staining time = 60 minutes, staining temperature = 80 °C.

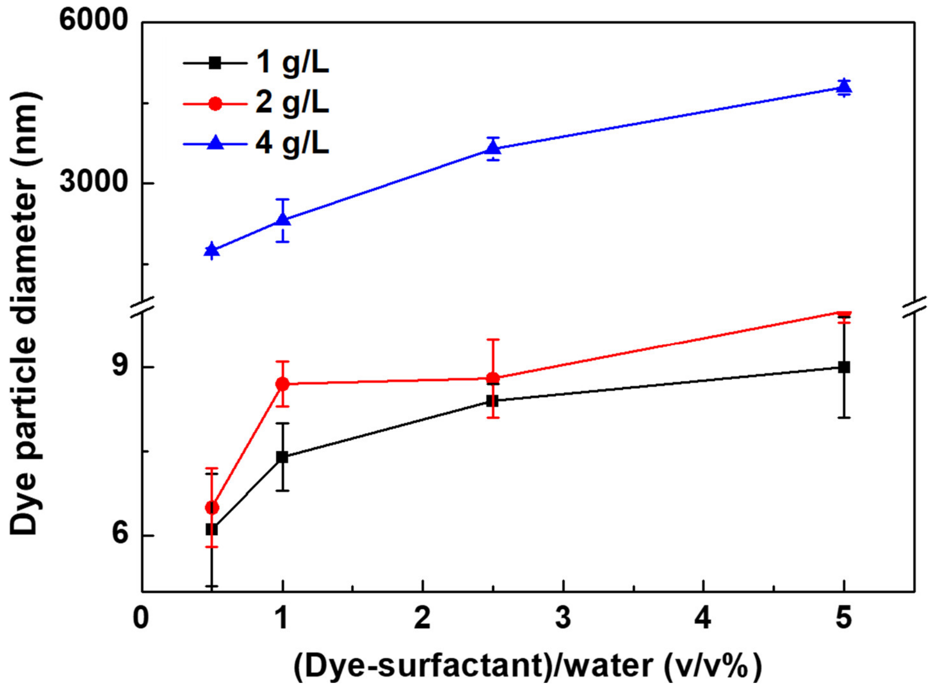

Figure S2. Size of the dye particles with different dye/surfactant ratios at various S-FN/Tween 20 concentrations in water.

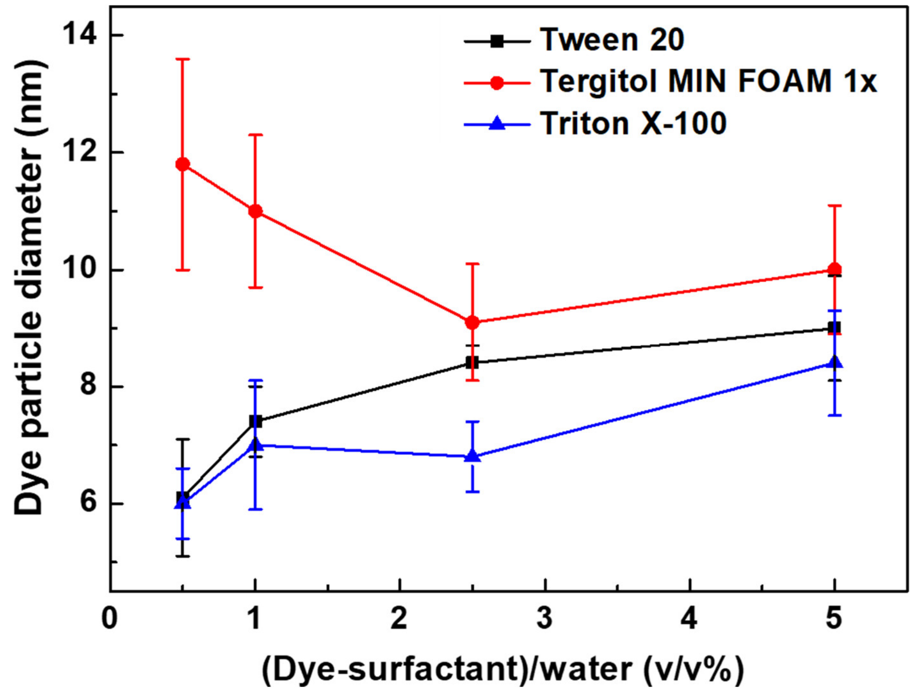

Figure S3. Size of the dye particles with different surfactants at various S-FN concentrations in water (dye/surfactant = 1 g/L).

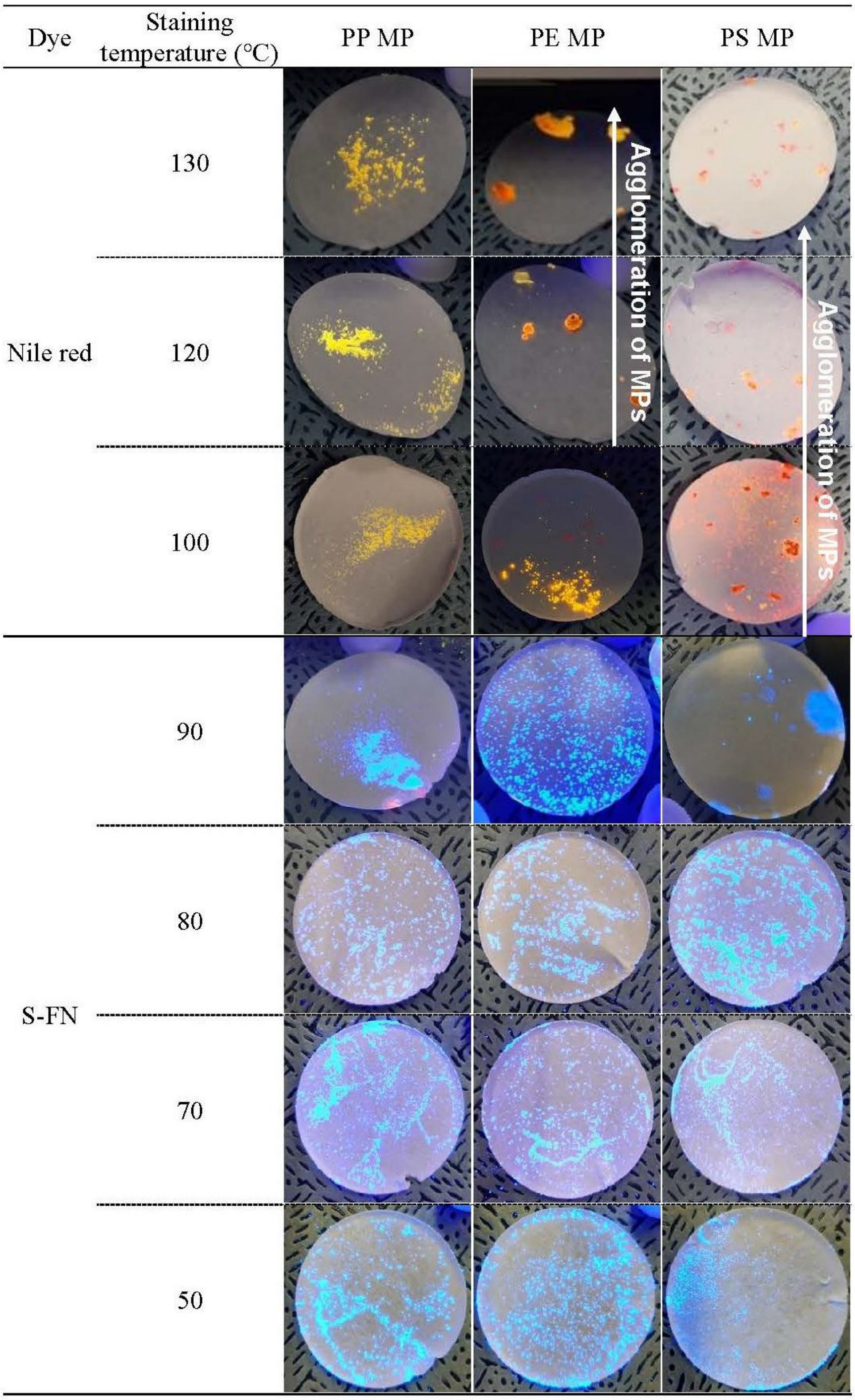

Figure S4. Optical photographs of different stained MPs at different staining temperatures (illumination by UV Hand Lamp at 254 nm for MPs stained with Nile red and 365 nm for MPs stained with S-FN). Staining conditions: surfactant = Tween 20, dye/surfactant = 1 g/L, dye/MP = 2.5 wt%, MP/aqueous solution = 1 g/L, staining time = 60 minutes.

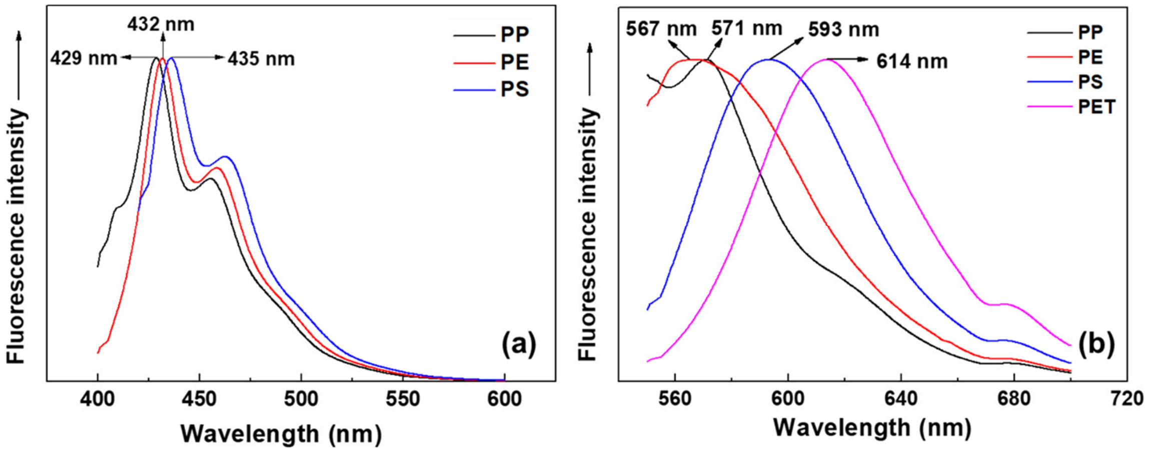

Figure S5. Normalized fluorescence spectra of S-FN (a) and Nile red (b) in stained MPs. Staining conditions: surfactant = Tergitol MIN FOAM 1x, dye/surfactant = 1 g/L, dye/MP = 2.5 wt%, MP/aqueous solution = 1 g/L, staining time = 60 minutes, staining temperature = 80 °C.

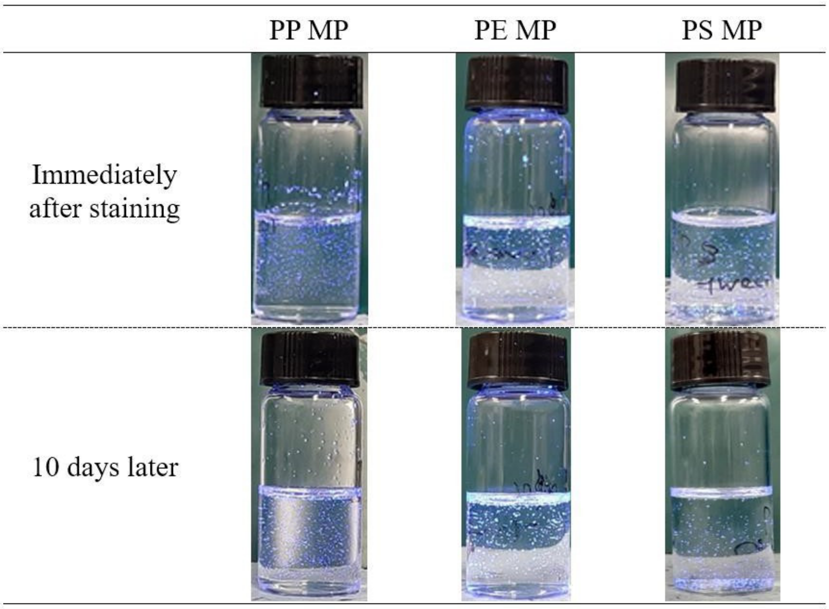

Figure S6. Optical photographs of stained MPs after removing the staining solution and redispersing the MPs in a water/ethanol solution (illuminated by UV Hand Lamp at 365 nm). Staining conditions: dye = S-FN, surfactant = Tween 20, dye/surfactant = 1 g/L, dye/MP = 2.5 wt%, MP/aqueous solution = 1 g/L, staining time = 60 minutes, staining temperature = 80 °C.

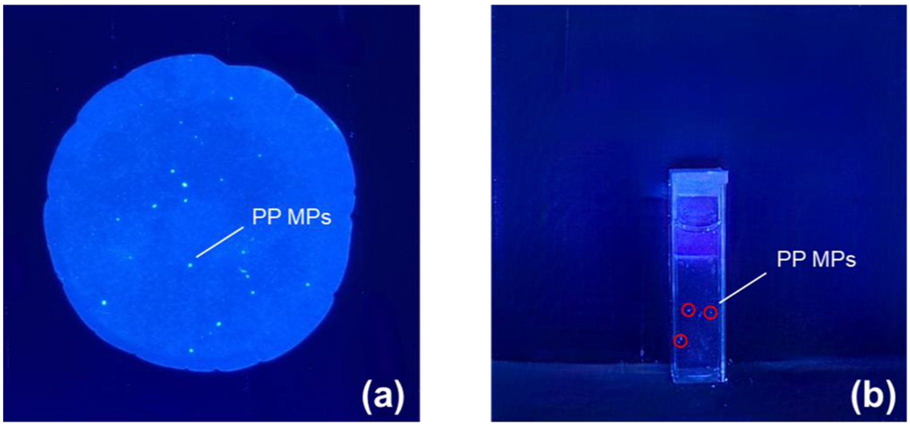

Figure S7. Optical photographs of PP MPs stained at an MP/aqueous solution concentration of 3.5 × 10^{-2} g/L: Filtered PP MPs (a) and redispersed PP MPs in aqueous solution (b) after staining (illuminated by UV Hand Lamp at 365 nm). Staining conditions: dye = S-FN, surfactant = Tween 20, dye/surfactant = 1 g/L, dye/MP = 2.5 wt%, MP/aqueous solution = 3.5 × 10^{-2} g/L, staining time = 60 minutes, staining temperature = 80 °C.

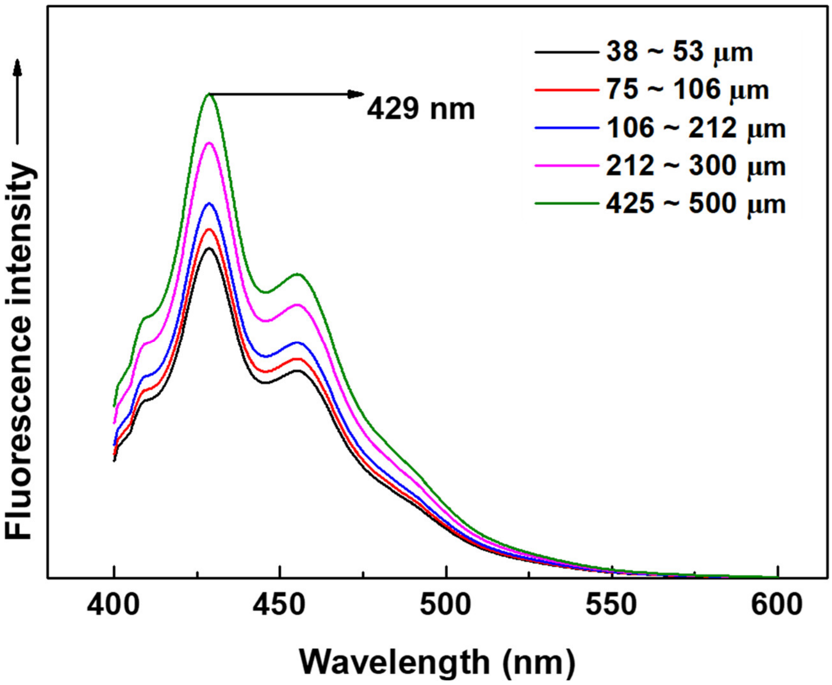

Figure S8. Fluorescence spectra of PP MPs with different sizes stained with S-FN/Tween 20. Staining conditions: dye/surfactant = 1 g/L, dye/MP = 2.5 wt%, MP/aqueous solution = 1 g/L, staining time = 60 minutes, staining temperature = 80 °C.

References

1. Karakolis, E.G.; Nguyen, B.; You, J.B.; Rochman, C.M.; Sinton, D. Fluorescent dyes for visualizing microplastic particles and fibers in laboratory-based studies. *Environ. Sci. Technol. Lett.* **2019,** *6*, 334-340.
2. Maes, T.; Jessop, R.; Wellner, N.; Haupt, K.; Mayes, A.G. A rapid-screening approach to detect and quantify microplastics based on fluorescent tagging with Nile Red. *Sci. Rep.* **2017,** *7*, 44501.
3. Tamminga, M.; Hengstmann, E.; Fischer, E.K. Nile Red staining as a subsidiary method for microplastic quantification: A comparison of three solvents and factors influencing application reliability. *SDRP J. Earth Sci. Environ. Studies* **2017,** *2*, 165-172.
4. Hengstmann, E.; Fischer, E.K. Nile red staining in microplastic analysis-proposal for a reliable and fast identification approach for large microplastics. *Environ. Monit. Assess.* **2019,** *191*, 612.
5. Erni-Cassola, G.; Gibson, M.I.; Thompson, R.C.; Christie-Oleza, J.A. Lost, but found with Nile red: A novel method for detecting and quantifying small microplastics (1 mm to 20 μm) in environmental samples. *Environ. Sci. Technol.* **2017,** *51*, 13641-13648.

6. Gagne, F.; Auclair, J.; Quinn, B. Detection of polystyrene nanoplastics in biological samples based on the solvatochromic properties of Nile red: application in *Hydra attenuata* exposed to nanoplastics. *Environ. Sci. Pollut. Res.* **2019,** *26*, 33524-33531.
7. Lv, L.; Qu, J.; Yu, Z.; Chen, D.; Zhou, C.; Hong, P.; Sun, S.; Li, C. A simple method for detecting and quantifying microplastics utilizing fluorescent dyes - Safranine T, fluorescein isophosphate, Nile red based on thermal expansion and contraction property. *Environ. Pollut.* **2019,** *255*, 113283.
8. Stanton, T.; Johnson, M.; Nathanail, P.; Gomes, R.L.; Needham, T.; Burson, A. Exploring the efficacy of Nile red in microplastic quantification: A costaining approach. *Environ. Sci. Technol. Lett.* **2019,** *6*, 606-611.
9. Tong, H.; Jiang, Q.; Zhong, X.; Hu, X. Rhodamine B dye staining for visualizing microplastics in laboratory-based studies. *Environ. Sci. Pollut. Res.* **2021,** *28*, 4209-4215.
10. Wiggin, K.J.; Holland, E.B. Validation and application of cost and time effective methods for the detection of 3-500 μm sized microplastics in the urban marine and estuarine environments surrounding Long Beach, California. *Mar. Pollut. Bull.* **2019,** *143*, 152-162.
11. Shim, W.J.; Song, Y.K.; Hong, S.H.; Jang, M. Identification and quantification of microplastics using Nile Red staining. *Mar. Pollut. Bull.* **2016,** *113*, 469-476.
12. Tosic, T.N.; Vruggink, M.; Vesman, A. Microplastics quantification in surface waters of the Barents, Kara and White Seas. *Mar. Pollut. Bull.* **2020,** *161*, 111745.
13. Cook, S.; Chan, H.L.; Abolfathi, S.; Bending, G.D.; Schafer, H.; Pearson, J.M. Longitudinal dispersion of microplastics in aquatic flows using fluorometric techniques. *Water Res.* **2020,** *170*, 115337.
14. James, E. M., *Polymer Data Handbook*. Oxford University Press: New York, 1999.
15. Merrington, A., 11 - Recycling of Plastics. In *Applied Plastics Engineering Handbook*, Kutz, M., Ed. William Andrew Publishing: Oxford, 2011; pp 177-192.
16. Adachi, K.; Takahashi, T.; Kamehashi, K.; Watanabe, K.; Uchiyama, K.; Kuriyama, T.; Miyata, K.; Hisamastu, T. Thermal Effects on Ultrasonic Joining of Thin Plastic Films Using Torsional Vibrations. *Jpn. J. Appl. Phys.* **2008,** *47*, 6431-6436.
17. Gent, M.; Sierra, H.M.; Menendez, M.; de Cos Juez, F.J. Evaluation of ground calcite/water heavy media cyclone suspensions for production of residual plastic concentrates. *Waste Manag* **2018,** *71*, 42-51.
18. Liu, T.-W.; Youngstrom, C.R.; Agarwal, S.; Al-Mulla, A.; Gaggar, S.K.; Gupta, R.K., Novel Biomass-Based Non-Halogenated FR Styrenic Blends. In *Fire and Polymers VI: New Advances in Flame Retardant Chemistry and Science*, 2012; pp 151-165.
19. Andrady, A.L. Microplastics in the marine environment. *Mar. Pollut. Bull.* **2011,** *62*, 1596-1605.
20. Prata, J.C.; da Costa, J.P.; Duarte, A.C.; Rocha-Santos, T. Methods for sampling and detection of microplastics in water and sediment: A critical review. *Trends Anal. Chem.* **2019,** *110*, 150-159.
21. Andrady, A.L. The plastic in microplastics: A review. *Mar. Pollut. Bull.* **2017,** *119*, 12-22.
22. Hidalgo-Ruz, V.; Gutow, L.; Thompson, R.C.; Thiel, M. Microplastics in the marine environment: a review of the methods used for identification and quantification. *Environ. Sci. Technol.* **2012,** *46*, 3060-3075.
